# Minimally invasive treatment of urinary fistulas using N-butyl-2-cyanoacrylate: a valid first line option

**DOI:** 10.1186/1471-2490-13-55

**Published:** 2013-10-24

**Authors:** Cesare Selli, Maurizio De Maria, Michele Manica, Filippo Maria Turri, Francesca Manassero

**Affiliations:** 1 Department of Urology, University of Pisa, Pisa, Italy; 2 Urologia Universitaria, Edificio 30 C, via Paradisa 2, Pisa, I-56124, Italy

**Keywords:** Urinary fistulas, N-butyl-2-cyanoacrylate

## Abstract

**Background:**

A few single case reports and only one clinical series have been published so far about the use of N-butyl-2-cyanoacrylate in the treatment of urinary fistulas persisting after conventional urinary drainage.

**Case presentation:**

We treated five patients with a mean age of 59.2 years presenting iatrogenic urinary fistulas which persisted following conventional drainage manouvres. There were 3 calyceal fistulas following open, laparoscopic and robotic removal of renal lesions respectively, one pelvic fistula after orthotopic ileal neobladder and a bilateral dehiscence of uretero-sigmoidostomy. We used open-end catheters of different sizes adopting a retrograde endoscopic approach for cyanoacrylate injection in the renal calyces, while a descending percutaneous approach via the pelvic drain tract and bilateral nephrostomies respectively was used for the pelvic fistulas. Fluoroscopic control was always used during the occlusion procedures. The amount of adhesive injected ranged between 2 and 5 cc and in one case the procedure was repeated. With a median follow-up of 11 months we observed clinical and radiological resolution in 4 cases (80%), while a recurrent and infected calyceal fistula after laparoscopic thermal renal damage during tumor enucleoresection required nephrectomy. No significant complications were documented.

**Conclusions:**

In an attempt to spare further challenging surgery in patients that had been already operated on recently, minimally invasive occlusion of persistent urinary fistulas with N-butyl-2-cyanoacrylate represents a valid first line treatment, justified in cases when the urinary output is not excessive and there is a favorable ratio between the length and diameter of the fistulous tract.

## Background

The conventional treatment algorithm for iatrogenic urinary fistulas previews first an adequate urine drainage via percutaneous nephrostomies, ureteral double J stents and/or indwelling bladder catheter, according to their location. If these maneuvers fail, occlusion of the fistulous tract can be attempted, since surgical repair may be challenging. Biologic agents like thrombin, fibrin, collagen glues have been successfully used for over one decade, adopting either a percutaneous or a retrograde endoscopic approach [1,2]. More recently the application of cyanoacrylate glues has been reported in different branches of surgery [3-5] and also for the management of urinary fistulas. In particular a sealant (Glubran 2™) composed of N-butyl-2-cyanoacrylate monomer and metacryloxysulpholane monomer has been used in the only clinical series published so far with a high success rate and a few complications [6]. This compound presents favorable properties such as a good biocompatibility and progressive reabsorption without causing foreign body granulomas. Its polymerization time is rather fast (beginning after 5 seconds and terminating after 90 seconds) in a wet environment, and water actually acts as a catalyst [7]. We present our experience with five cases of persistent iatrogenic urinary fistulas where minimally invasive treatment with this synthetic glue was employed, in an attempt to spare further challenging surgery in patients that had been already operated on recently.

## Case presentation

**Figure 1:**
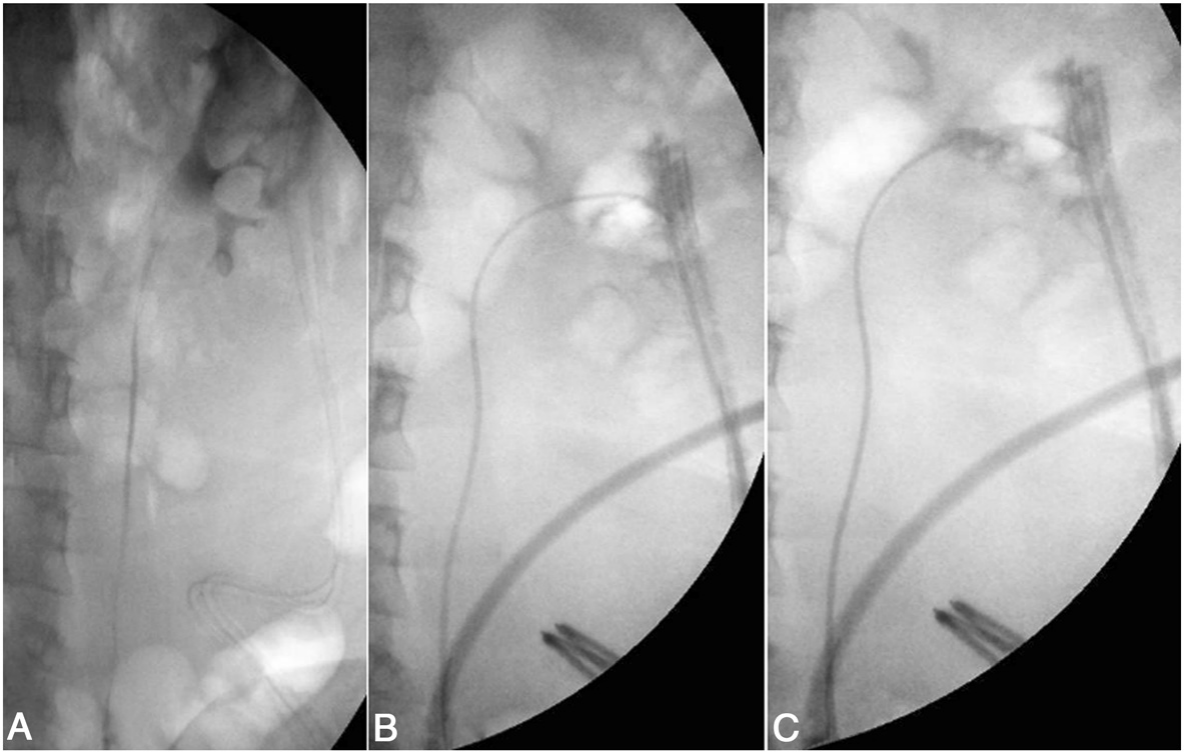
**Treatment of case n. 4. A)** retrograde pyelography revealing perirenal extravasation of contrast medium after robot assisted enucleoresection of RCC. **B)** An open-end 5 F Cobra catheter is advanced in the middle calyx in preparation for embolization of the calyceal fistula. **C)** Retrograde contrast injection following embolization with cyanoacrylate.

During the period 2002–2013 a cohort of 5 patients ranging in age between 48 and 73 years (mean 59.2), were hospitalized for the treatment of iatrogenic fistulas which failed to resolve after conventional urine drainage. The relevant demographic, clinical and follow-up data are reported in Table 1. There were 3 calyceal fistulas occurring respectively after open removal of complex hemorrhagic renal cysts, laparoscopic enucleoresection and robot-assisted enucleoresection for pT1 renal cell carcinomas. Following documentation of urine leak though the drainage tube, the initial treatment had consisted in all in endoscopic placement of a double J stent and gravity drainage of the bladder via an indwelling Foley catheter, but after a median period of 32 days urine leak persisted, and in one patient it became grossly infected. Under fluoroscopic guidance the double J stents were endoscopically removed and an open-end 6 F ureteral catheter (Boston Scientific) was advanced over an hydrophilic guide wire in the upper calyx in 2 cases, while and angled 5 F Cobra catheter (Cordis) was advance in the middle posterior calyx in the third patient, placing the tip close to the fistula site. An amount of cyanoacrylate sealant between 2 and 5 cc was injected and the ureteral catheter was rapidly withdrawn. After a few minutes a new open-end ureteral catheter was advanced in the renal pelvis, and a control retrograde pyelography was performed (Figure 1). Finally a new double J ureteral stent was placed and the bladder was drained with a Foley catheter. In two cases there was immediate resolution of urine leak through the lumbar drainage, which was removed after 24 hours. In one patient with persisting drainage, following evidence at uro-CT scan of contrast medium outside the kidney, the procedure was repeated after 15 days. All the endoscopic maneuvers were performed in the operating room under light sedation and were well tolerated.

**Table 1:** Demographic and clinical data of 5 patients treated with occlusion of urinary fistulas using cyanoacrylate

**Patient**	**Sex**	**Age**	**Diagnosis**	**Persistent drainage days**	**Control imaging**	**Follow-up months**	**Access**	**Outcome**
1	M	63	Neobblader-cutaneos fistula	90	Cystography	38	Percutaneous trough drainage sinus	Cured
2	M	73	Bilateral dehiscence of ureterosigmoidostomy	32	Bilateral trans-nephrostomic pyelography	3	Percutanuous bilateral trans-nephrostom	Cured
3	F	62	Calyceal fistula after open removal of complex renal cyst	10	Retrograde pyelography	103	Retrograde endoscopic	Cured
4	F	50	Calyceal fistula after robot-assisted enucleoresection of RCC	13	Retrograde pyelography CT scan	11	Retrograde endoscopic	Cured
5	M	48	Calyceal fistula after laparoscopic enucleoresection of RCC	38	Retrograde pyelography CT scan	3	Retrograde endoscopic (repeated)	Failure - Nephrectomy
		Mean 59.2		Mean 36.6		Mean 31.6		
				Median 32		Median 11		

**Figure 2:**
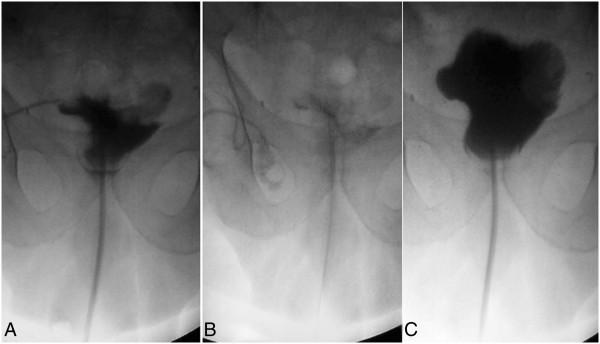
**Treatment of case n. 1. A)** Retrograde opacification of orthotopic neobladder revealing a fistula through the right pelvic drain. **B)** Fistula occlusion using an open-end 7 F catheter advanced through the fistulous tract. **C)** Retrograde opacification of the neobladder 24 hours later showing fistula closure.

We treated a neovesico-cutaneous fistula following radical cystectomy and Studer orthotopic neobladder, with urinary drainage through the right pelvic drain which persisted 3 months in spite of indwelling bladder catheter. In the radiology suite a guidewire was advanced inside the drain, which was removed and exchanged with a 7 F open-end catheter, through which the mature fistulous tract was embolized with 3 cc of Glubran2. After cystographic control demonstrating tract closure, the Foley catheter in the neobladder was removed after 24 hours (Figure 2).

The last patient presented advanced sclerodermia and a pT3N1M0 bladder cancer treated with radical cystectomy and sigmoid ureterostomy, since any form of external urinary drainage was unadvisable due to the skin condition, and the patient was unfit for orthotopic neobladder. Urinary drainage through both the pelvic drains became apparent in the 9th postoperative day, due to bilateral dehiscence of the uretero-intestinal sutures stented with transanal Bracci PVC catheters (Porgès, France), presumably due to vasculitis. Bilateral percutaneous nephrostomies failed to improve the urinary leak, and after 12 days in the radiology suite the Bracci catheters were withdrawn transanally, two open-end 6 F catheters were advanced antegradely in the middle uterers and embolization was performed on each side with 2 cc of cyanoacrylate sealant, determinig successful ureteral occlusion and leaving two permanent percutaneous nephrostomies. Due to progressive deterioration of the general condition the patient deceased 3 months later.

**Figure 3:**
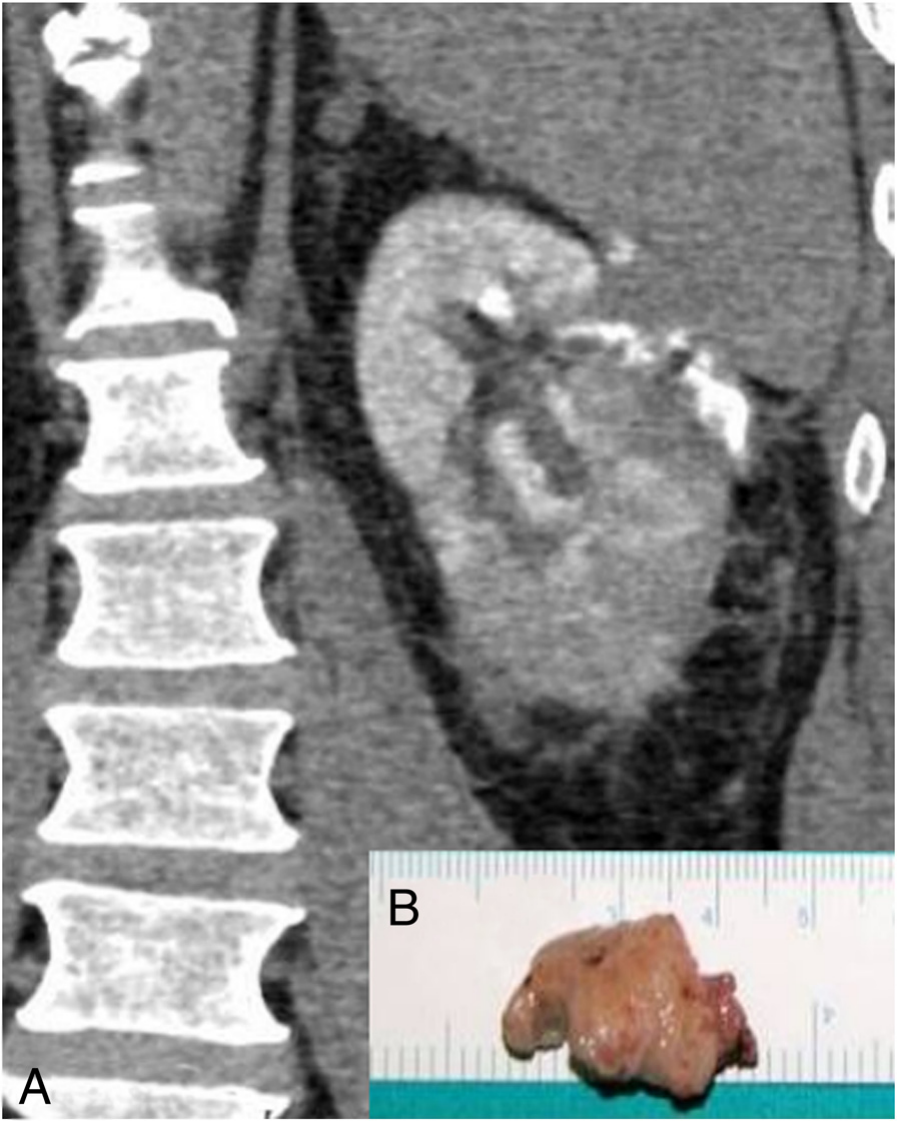
**Treatment of case n. 5. A)** Uro-CT scan following repeated embolization of a fistula of the upper calyx caused by thermal damage during laparoscopic enucleoresection of RCC. The radiopaque urine flows around the calyceal plug and reaches the external kidney surface. **B)** Inset: cyanoacrylate cast of the upper calyx removed at nephrectomy.

Median follow-up was 11 months (range 3–103), and no significant complications were observed after the occlusion procedure, in particular there was no secondary stone occurrence. The occlusion procedure was successful in 4 out of 5 cases (80%) and the only patient where it failed, after being repeated, presented extensive thermal renal damage determined during laparoscopic tumor enucleoresection. This resulted in a high-output calyceal fistula through the lumbar drain, which later became infected. For this reason and for patient choice a nephrectomy was performed, and on the operative specimen there was cyanoacrylate adherent to the external kidney surface and a 25 × 18 mm plug in the upper calyx, but evidently urine continued to flow around it (Figure 3). The 3 surviving patients where the procedure was successfully performed have been regularly controlled clinically and with imaging techniques.

Iatrogenic urinary tract fistulas may present as early complications following urological and gynecological procedures, and their incidence is likely to increase due to the diffusion of laparoscopic and robotic surgery, where inadvertent thermal damage to the kidney and ureter by different energy sources may cause urine leak. Their conventional management consists in appropriate urinary drainage, which can be sufficient for tissue healing and fistula closure, provided that the amount of leakage is not excessive and the tissue damage is limited. Surgical repair is advisable when there is no improvement after about 3 months, but this view has been challenged for vesicovaginal and vesicouterine fistulas, where an earlier treatment may be advisable. The interval period is generally source of considerable stress for the patients and malpractice suits may potentially arise. On the other hand open fistula repair may be technically challenging and a decreased renal function is to be considered as the final result. In particular calyceal fistulas for adequate repair often require partial or total nephrectomy.

In the early postoperative period therefore there is room for attempts at fistula closure using minimally invasive techniques, and cyanoacrylate sealants offer new possibilities in this field. Until now there are only 5 single case reports [8-12], which obviously had all a favorable outcome, besides the only sizable clinical series of 13 cases cumulated in 9 years, documenting a success rate of 84.6% with a median follow-up of 35 months [6]. There is also an interesting recent report of minimally invasive treatment of 10 patients presenting persistent or massive urine leaks at the vescicorethral anastomosis following radical prostatectomy using cystoscopically cyanoacrylate mixed with lipiodol to fill retrogradely the anastomotic gap, followed by apposition of fibrin glue in direct contact with urine [13]. This combination of sealants was used with the purpose of avoding a rigid and potentially lithogenic material at the level of the anastomosis, which could impair urinary continence recovery and cause strictures. However the reported incidence of secondary lithiasis using cyanoacrylates is low, with only one case of bladder stone following occlusion of vesico-sigmoid fistula [6].

In our experience the presence of small granules of polymerized cyanoacrylate inside the upper urinary following embolization of calyceal fistulas was not harmful, since they were spontaneously eliminated along the double J stents. However in the patient were a nephrectomy was necessary following 2 attempts at fistula closure, we found a cyanoacrylate cast of the upper calyx, where potentially encrustation could have occurred. It is also theoretically possible that a sizable fragment of the sealant could be dislodged after removal of the double j stent, causing ureteral occlusion.

To the best of our knowledge all the 5 reported cases of calyceal fistula embolization have been perfomed percutaneously [6,8-11], while in our opinion, unless a percutaneous nephrostomy already exists, a retrograde endoscopic approach is justified, since it is less invasive. We successfully occluded with this approach 2 out of 3 calyceal fistulas.

In our experience, as well in that of the other clinical series published [6], the critical factor for success in fistula closure using cyanoacrylate is the ratio between the length and the diameter of the fistulous tract, since tissue losses greater than 1 cm and short are unlikely to be completely plugged, and urine may continue to flow around the cast. The amount of urine drained daily and the presence of concomitant infection are likely to represent also key factors, as well of the location of the fistulous tract.

In case 3, where repeated embolization of calyceal fistula was unsuccessful, both the wide size of the fistulous tract and concomitant bacterial infection were in our opinion factors contributing to treatment failure and subsequent kidney removal.

## Conclusions

In our experience a cyanoacrylate-based sealant, originally proposed for interventional radiology, was also suitable for occlusion of urinary fistulas in different locations using both percutaneous and endoscopic approaches. Since this treatment is well tolerated, without major complications and minimally invasive, in our opinion it is worthwhile to consider it relatively early when conventional urinary drainage measures fail. By doing so considerable stress can be avoided to the patients.

The fistula diameter to length ratio is the more relevant prognostic factor for success, but since this sealant causes minimal tissue reaction, it does not affect significantly subsequent open surgical repair. Our encouraging results need to be validated by other studies, but the uncommon occurrence of persistent urinary fistulas and the fact that the use of cyanoacrylate inside the urinary tract is not universally feasible and approved are factors explaining the relative rarity of clinical series concerning this minimally invasive treatment.

## Consent

Written informed consent was obtained from the patients for the publication of this report and any accompanying images.

## Competing interests

The authors declare that they have no competing interests.

## Authors’ contributions

CS ideated the study. FM, MM acquired the data. FMT drafted the manuscript. MDM, CS treated the patients. CS, FM critically revised the manuscript. All authors read and approved the final manuscript.

## Pre-publication history

The pre-publication history for this paper can be accessed here:

http://www.biomedcentral.com/1471-2490/13/55/prepub
